# AlphaFold, small-angle X-ray scattering and ensemble modelling: a winning combination for intrinsically disordered proteins

**DOI:** 10.1107/S1600576723008403

**Published:** 2023-09-27

**Authors:** Véronique Receveur-Bréchot

**Affiliations:** a CNRS, Aix Marseille Univ., BIP Bioénergétique et Ingénierie des Protéines, UMR7281, F-13402 Marseille, France

**Keywords:** small-angle X-ray scattering, SAXS, AlphaFold, ensemble modelling, structural flexibility

## Abstract

A recent article by Brookes, Rocco, Vachette & Trewhella [*J. Appl. Cryst.* (2023), **56**, 910–926] on improving the accuracy of AlphaFold structural predictions for disordered proteins is discussed.

Artificial intelligence has revolutionized many societal and scientific domains, and structural biology benefits from one of its most spectacular breakthroughs. The deep learning algorithm AlphaFold can predict with unparalleled accuracy the three-dimensional structure of folded proteins based solely on their sequence. In 1972, Christian Anfinsen was awarded the Nobel Prize in Chemistry for his groundbreaking work on ribonuclease which demonstrated that all the protein’s folding information is encoded within its sequence. This ignited a surge of experimental and computational investigations into protein folding to unravel what was at the time referred to as the ‘second half of the genetic code’ and understand the mechanisms governing the protein’s pathway towards its final three-dimensional conformation.

Now, half a century after Anfinsen’s seminal article (Anfinsen, 1973 ▸), it appears that a long-awaited milestone has been reached and that one can now correlate a one-dimensional string of amino acids to an accurate three-dimensional structure. However, challenges still persist. The fundamental nature of artificial intelligence lies in its initial training using data that have accumulated over many years, the Protein Data Bank (PDB) in the case of AlphaFold. The PDB suffers from inherent biases: certain families of structures are under-represented due to the limitations of the methods used to solve the protein structures, in particular X-ray crystallization and cryo-electron microscopy (cryoEM). This is typically the case of proteins that exhibit a high conformational flexibility and cannot be described by a single three-dimensional structure. In particular, intrinsically disordered proteins (IDPs) or regions (IDRs) of proteins can adopt a tremendous number of conformations and this high dynamic is inherent to their function. Because of their flexibility, these polypeptides are often invisible in the structures solved by X-ray crystallization or cryoEM. Their flexibility may even preclude protein crystallization or lead to poorly diffracting crystals. In addition, a short disordered segment can undergo an induced folding upon binding to a partner, but may adopt different folds depending on the binding partner and the interface of interaction, further complicating accurate predictions. Therefore, AlphaFold could not train properly on disordered structures, which lack structural homologues in the PDB. Furthermore, AlphaFold uses multiple sequence alignments, while IDRs are very difficult to align because they are poorly conserved. For all these reasons AlphaFold cannot predict for now the 3D structure of long flexible disordered regions with accuracy and provides low confidence scores on these predictions. AlphaFold’s creators even suggest that the low confidence scores can be used as a method to predict protein disorder. Therefore, the single static representation of the structure of proteins containing IDRs provided by AlphaFold cannot convey all their structural and functional properties.

A very interesting strategy to circumvent this difficulty is to integrate AlphaFold predictions with ensemble molecular modelling and experimentally derived constraints, as proposed by Brookes *et al.* (2023 ▸) in their paper entitled *AlphaFold-predicted protein structures and small-angle X-ray scattering: insights from an extended examination of selected data in the Small-Angle Scattering Biological Data Bank*. Small-angle X-ray scattering (SAXS) is a particularly suitable experimental method for providing such constraints. SAXS data contain all the 3D information on the various conformations adopted by a macromolecule in solution. By calculating the SAXS profile from atomic coordinates and comparing it with experimental SAXS data, it becomes possible to select an ensemble of conformations that collectively contribute to the observed scattering spectrum. These conformers are generated through molecular modelling techniques that allow incorporation of information on the protein flexibility into the predicted structures.

To prove the effectiveness of this strategy, Brookes and co-workers utilized the Small Angle Scattering Biological Data Bank (SASBDB) as an additional resource. They selected monomeric AlphaFold models with a corresponding SASDBD entry, where the single AlphaFold model did not account for the collected SAXS data. The candidate proteins are composed of several folded domains connected by at least one long extended linker of more than 30 amino acids. These linkers are predicted to be unstructured by AlphaFold, with a low confidence score. Brookes and co-workers considered these regions as flexible and generated an ensemble of structures by exploring the conformational space of these regions using a Monte Carlo approach. They then selected the optimal ensemble of conformers and thus reached remarkable agreements with the SAXS data. Hence, by taking into account the conformational heterogeneity of the linkers, guided by the SAXS experimental data, they significantly improved the accuracy of the AlphaFold structural predictions (Fig. 1 ▸).

Besides the remarkable improvement in the structural description of the disordered domains, this article is striking for its rigorous analysis of the SAXS data. The authors are long-standing experts in small-angle scattering for structural biology, and they know all the strengths and limits of the technique (Trewhella *et al.*, 2022 ▸, 2017 ▸). In this new paper, Brookes, Rocco, Vachette and Trewhella explain with a lot of teaching the methodology, quality assessment, pitfalls and difficulties of SAXS data analysis that need to be known before any atomistic modelling, with an emphasis on the specific case of proteins with long IDRs. Their thorough approach and their valuable advice will undoubtedly inspire and guide structural biologists struggling with SAXS experiments on IDPs, enabling them to confidently extract all the relevant information on their favourite protein from their data.

Finally, this paper successfully addressed the limitations of AlphaFold in predicting the structure of proteins with long IDRs, by incorporating adequate conformational flexibility. While AlphaFold has enabled considerable advances in structural biology, its next challenge is to accurately predict the conformations preferentially adopted by IDPs and IDRs. Efforts are currently being made to develop accurate predictions of conformational ensembles using machine learning with reduced computational cost (Zheng *et al.*, 2023 ▸). However, the conformational sampling of IDPs or IDRs is extremely sensitive to the environmental conditions, such as pH, temperature, redox conditions, crowding, ligand binding, post-translational modifications *etc*., and this is the motor of their activity. Experimentally derived constraints accounting for these conditions therefore remain necessary to accurately describe the dynamics and structural preferences that drive the functional properties of an IDP. SAXS, being one of the few experimental techniques that give access to the conformational sampling of proteins in solution, combined with ensemble modelling and AlphaFold predictions offers a promising strategy for adequately describing the structure and dynamics of IDPs and is expected to yield numerous successful advances in the understanding of these proteins.

## Figures and Tables

**Figure 1:**
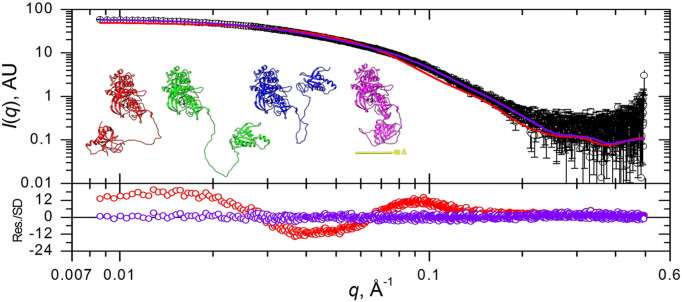
The fit to the experimental SAXS data is significantly improved when an ensemble of conformers is used (purple line) compared with the single predicted AlphaFold model (red line). Figure adapted from Brookes *et al*. (2023 ▸).
